# HYADES - A Global Archive of Annual Maxima Daily Precipitation

**DOI:** 10.1038/s41597-024-03109-2

**Published:** 2024-03-15

**Authors:** Mijael Rodrigo Vargas Godoy, Simon Michael Papalexiou, Yannis Markonis

**Affiliations:** 1 Faculty of Environmental Sciences, Czech University of Life Sciences Prague, Kamýcká 129, 165 00 Praha-Suchdol, Czech Republic; 2 Schulich School of Engineering, University of Calgary, Calgary, Canada; 3 Global Institute for Water Security, University of Saskatchewan, Saskatoon, Canada

**Keywords:** Hydrology, Hydrology

## Abstract

Time series of annual maxima daily precipitation are crucial for understanding extreme precipitation behavior and its shifts toward nonstationarity with global warming. Extreme precipitation insight assists hydraulic infrastructure design, water resource management, natural hazard prevention, and climate change adaptation. However, not even a third of the records are of sufficient length, and the number of active stations keeps decreasing. Herein, we present HYADES: archive of yearly maxima of daily precipitation records, a global dataset derived from the Global Historical Climatology Network database of daily records (GHCN-Daily). The HYADES dataset contains records from 39 206 stations (heterogeneously distributed worldwide) with record lengths varying from 16 to 200 years between 1805 and 2023. HYADES was extracted through a methodology designed to accurately capture the true maxima even in the presence of missing values within the records. The method’s thresholds were determined and evaluated through Monte Carlo simulations. Our approach demonstrates a 96.73% success rate in detecting the true maxima while preserving time series statistical properties of interest (L-moments and temporal monotonic trend).

## Background & Summary

Precipitation characteristics (e.g., duration, frequency, and intensity) are expected to transmute with climate change^1^. Out of these changing characteristics, understanding the intensity of precipitation extremes, which is expected to shift towards nonstationarity^2^, is particularly important due to its applicability in fields like risk assessment of floods^3^ and landslides^4^, as well as the design of hydraulic engineering projects in general^5^ through concepts like the probable maximum precipitation (PMP) and the corresponding probable maximum flood (PMF)^6^. In other words, while the climatology, meteorology, hydroclimatology, and hydrology communities may come to mind as the main stakeholders of extreme precipitation data for research purposes, this information pertains to various other communities like civil engineers, urban planners, policymakers, and governmental officers, among others.

Robust analysis of extreme precipitation, which has been the cornerstone for the abovementioned applications since the introduction of Gumbel’s Statistics of Extremes^7^, requires long time series of data. However, the number of active stations keeps on decreasing despite its acknowledged importance and implications for other data sources^8^, which unavoidably limits the number of rain gauges with records of sufficient length. For example, Frich *et al*.^9^ assessed circa 3 000 station records from national climate archives to report that global land areas were increasingly affected by a significant change in climatic extremes during the second half of the 20th century. Note that even though 3000 stations account for less than 3% of available stations worldwide, most regional studies are limited to relying on tens to hundreds of stations at best, e.g.: Tank and Können^10^ assessed 202 daily precipitation series to determine that wet extremes increased over Europe in the 1946–99 period. Series with more than 20% missing or incomplete years in the analysis period were excluded from the analyses; Alexander *et al*.^11^ analyzed approximately 200 temperature and 600 precipitation stations, with near-complete data for 1901–2003 covering vast Northern Hemisphere midlatitudes (and parts of Australia for precipitation). The authors report a tendency toward wetter conditions throughout the 20th century; Shrestha *et al*.^12^ relied on historical records from 23 stations in Nepal to evaluate future projections and report that 100-year return period rainfall will become a 20 or 25-year return period rainfall; Ghanim *et al*.^13^ evaluated trends in extreme precipitation using 24 meteorological stations in the northern highlands of Pakistan to report a decline in the intensity, frequency, and extent of precipitation extremes.

On top of scarce data availability worldwide regarding sufficient record length and spatial distribution, another major problem is the presence of missing values within the station records because extreme value analysis depends on comprehensive and lengthy data sets to produce robust estimateshas. If one were to discard any incomplete years within a station record, the number and length of available records would be harshly reduced. When a station record is free of missing values, systematic errors aside, we can have confidence that the maximum value of each year is the actual maximum. However, in the presence of missing values within a given year, if we want to keep a value for the annual maxima, we must ensure that no significant biases are introduced. Throughout the literature, infilling missing value methods, either physical, statistical, or machine learning based, are abundant. In simple terms, most of these methods could be comprehended as weighted averages of existing observations^14^, and the main differences between them lie in how these weights are determined. Note that since the sample average cannot exceed the maximum value of the observations, infilling methods, to a larger or lesser extent, smooth out the time series and reduce variability. Mishandling missing values within a time series of extreme precipitation may lead to significant biases reverberating on hydraulic infrastructure design and natural hazard prevention strategies.

On the one hand, the lesser evil, overestimating extreme precipitation, would affect overall design and infrastructure costs. On the other hand, underestimation leads to infrastructure failure that may harm the surrounding environment, human life, and property downstream. Another significant concern of carelessly infilling missing values is the potential introduction of spurious trends that may distort the true nature of extreme precipitation patterns, whose changes have been shown to differ significantly from changes in mean precipitation^15^. Even though a meticulous selection of an infilling method that balances data completeness without compromising accuracy might seem like the most reasonable path to pursue^16^, we explored the possibility of identifying the annual maxima of daily precipitation even if assessing a partially incomplete record.

While the paramount importance of reliable annual maximum daily precipitation data is evident, and several regional studies have addressed its implications^9–13,17–23^, to the best of our knowledge, there is no global dataset readily available for the scientific community. Herein, we describe the generation of the “arcHive of Yearly mAxima of Daily prEcipitation recordS” (HYADES) dataset to provide the longest and most comprehensively available collection of station-based time series in spite of the presence of missing values. HYADES was constructed through a stochastic and statistical framework using the station inventory of the daily Global Historical Climatology Network (GHCN-Daily)^24^. The HYADES end product comprises records of annual maximum daily precipitation extracted from 39 206 stations with varying lengths from 16 to 200 years within the 1805–2023 period. The spatial distribution of the stations is quite heterogeneous around the world. Notwithstanding, the methodology introduced and the scripts shared could be extended to any data records that, for one or another reason, are not available within the GHCN-Daily database. Therefore, HYADES is critical to support hydraulic infrastructure design and climate change research in the context of extreme precipitation events to inform policy and decision-making.

## Methods

### Data

**Fig. 1:**
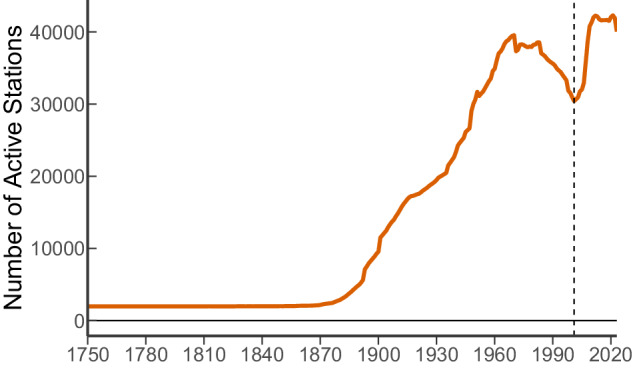
Time series of the number of active ground stations with total daily precipitation data as extracted from the Global Historical Climatology Network. The dashed lined denotes the year 2001 when the number of active ground stations had a trend inversion that lasted for a decade before reverting to an overall decreasing trend.

**Fig. 2:**
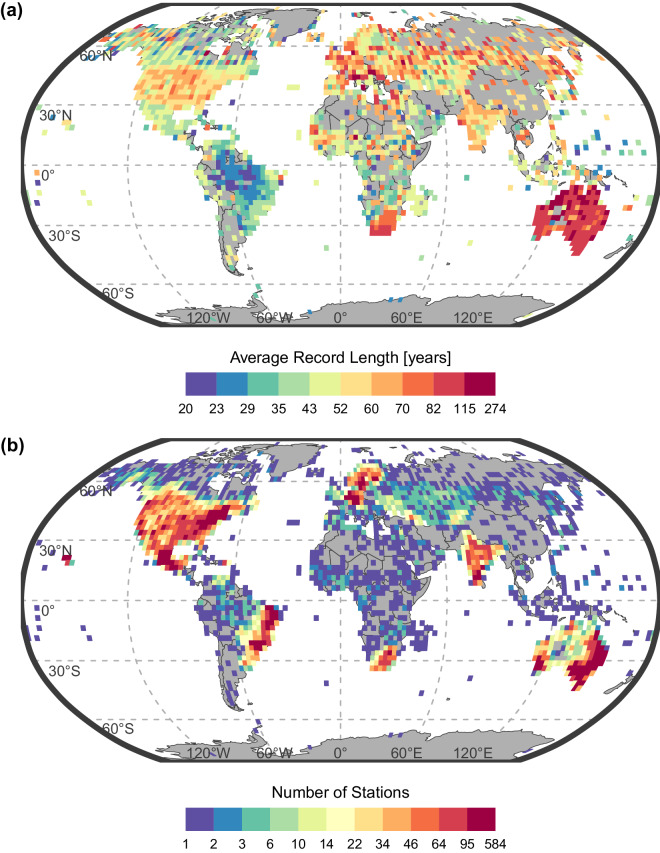
Spatial distribution of 43 585 ground stations with daily precipitation records of at least 20 years on a 2.5° × 2.5° regular grid. (**a**) Average record length of ground stations. (**b**) Number of ground stations per grid cell.

The Global Historical Climatology Network is managed by the National Centers for Environmental Information (NCEI), part of the National Oceanic and Atmospheric Administration (NOAA) of the United States of America. The GHCN-Daily database holds approximately 124 945 records of maximum and minimum temperature, total daily precipitation, snowfall, and snow depth from ground stations in 180 countries and territories^24^. Regarding those that contain total daily precipitation data, the oldest ground station records date back to 1781, and there are ground stations whose record start year is as recent as 2023. It is important to note that the number of active ground stations that had been continuously decreasing since the late 1960s had a relatively recent change point in 2001, reaching a maximum of 42 270 simultaneously active ground stations in 2012, and, regrettably, went back into a decreasing trend ever since (Fig. 1). Because of this new wave of active ground stations, we decided to include ground stations with a record length of at least 20 years instead of the 30 years required for the climate normal. Furthermore, by including ground stations whose record is as short as 20 years, we do not only increase their number but also expand their spatial coverage, as various of these recently deployed ground stations are located in previously uncharted regions. After this initial screening, we were left with 43 585 ground stations whose record length varies between 20 and 274 years (Fig. 2). Note that even by this preliminary step, before any analysis per se, we have already lost circa two thirds (65.12%) of the available ground station records held in the GHCN-Daily database. The fact that not even a third of the records are of sufficient length for analysis, in conjunction with the number of active ground stations decreasing during the past decade, should be an alarm and a call for action. Ground stations play a crucial role in collecting accurate and localized climate data, information that is essential for calibrating, evaluating, and enhancing the accuracy of other data sources like satellite remote sensing, climate models, and reanalyses.

The GHCN-Daily database undergoes rigorous quality control that involves identifying and correcting the collected information’s errors, inconsistencies, and outliers. Unfortunately, no matter how relentless the quality control is, this procedure mainly addresses data transmission problems, leaving a considerable number of ground station records with missing values caused by other factors like equipment malfunctions, sensor errors, or maintenance issues. Moreover, these missing values are not bound to be uniformly distributed over the entire record. For example, a given ground station record with a length of 100 years having 10% missing values does not entail that every year will have 0.1% missing values. Afterward, we scrutinized the missing value distribution profile in the selected ground station records to determine the best way to address incomplete years. Our investigation of the missing value profile includes:

1. The distribution of the record length in years for the selected ground stations (Fig. 3a). While the record lengths of the selected stations vary from 20 to 274 years, roughly half of the records have lengths shorter than 50 years, and the number of records remarkably drops for lengths longer than 80 years. Hence, most of the stations worldwide have a limited historical perspective.

**Fig. 3:**
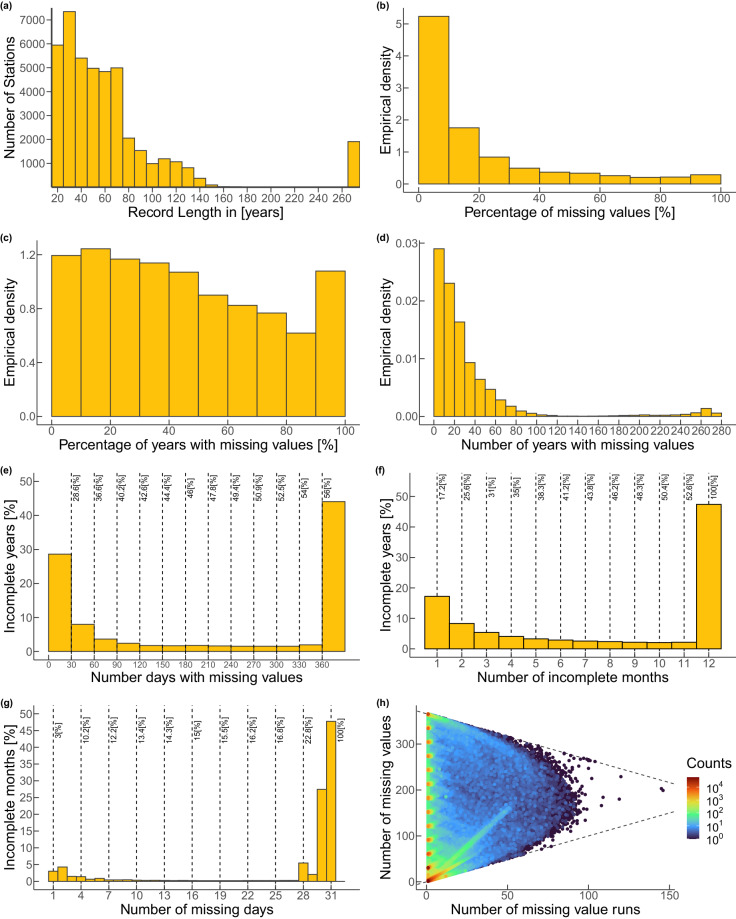
Missing value profile of the 43 585 selected stations. (**a**) Distribution of the record length in years. (**b**) Percentage of missing values in the records. (**c**) Percentage of years with missing values. (**d**) Number of years with missing values. (**e**) Number of missing days within incomplete years. (**f**) Number of moths with missing values within incomplete years. (**g**) Number of missing days within incomplete months. (**h**) Missing value structure within incomplete years.
2. The empirical distribution of the percentage of missing values within the selected ground stations (Fig. 3b). Note that since the records have a daily time step, a missing value corresponds to a missing day within the record. The majority of the records have less than 20% missing values. Albeit almost insignificant in comparison, there are records with more than 90% missing values. Even though it is out of scope herein, further inspection should be performed on the latter to understand the reasons behind such lofty levels of missing measurements.
3. The empirical distribution of percentage of incomplete years within the records (Fig. 3c). Herein, we define an incomplete year as any year within a record that has at least one missing value (i.e., an incomplete year has anywhere between one and 366 missing values), and the percentage of incomplete years is the ratio between the number of incomplete years and the record length in years. An evident inverse relation exists between the number of records and the percentage of incomplete years, but only between 10% and 90% below and above that range the inverse relation does not hold.
4. The empirical distribution of the number of incomplete years (Fig. 3d). Exploring the number of incomplete years within a record rather than its percentage, we confirm the previously observed inverse relation with the difference that it is not constrained to a range but extends to the whole range of values. The curve resembles an exponential decay. However, it must be noted that the distribution of record lengths biases this, i.e., it is impossible for a record to have more incomplete years than its record length.
5. The number of missing days given that a year is incomplete (Fig. 3e). The number of missing values appears to have a bimodal distribution with modes under 30 days and above 360, concentrating over 60% of the incomplete years. It would be hasty to affirm that given that a year is incomplete, it has either one month or the whole year missing because we only know the total number of missing days, not if they may or may not be consecutive.
6. The number of months with missing values within incomplete years (Fig. 3f). Herein, we define an incomplete month as any month within a record that has at least one missing value (i.e., an incomplete month has anywhere between one and 31 missing values). The number of incomplete months, just like the number of missing values, appears to have a bimodal distribution with modes at one month and 12 months, concentrating over 50% of the incomplete years. Given that a year is incomplete, a little under a third of them have 12 months incomplete.
7. The number of missing days within incomplete months (Fig. 3g). Given that a month is incomplete, two-thirds of them have more than 28 days missing, and less than a fifth of them have less than seven days missing. In other words, it is much more likely that a whole month is missing than only a few days within a month.
8. The missing value structure within incomplete years (Fig. 3h). Namely, the number of missing values versus the number of missing value runs. Herein, we define a missing value run as the chain of consecutive missing values before a non-missing value. As expected from the previous exploration, the darkest hotspot is a single run of 30 days. There are multiple other hotspots, albeit of an order of magnitude smaller, for single runs of multiple months (i.e., around 60, 90, 120, 150, 180, 210, 240, 270, 300, 330, and 360 days). In summary, missing values within them are more commonly observed in sequence than sporadically. Consequently, attempting to fill in the missing values before extracting the annual maxima would be meaningless.

### Threshold criteria

A preliminary method to extract the annual maxima from a daily time series containing missing values was proposed by Papalexiou and Koutsoyiannis^25^. Therein, the time series selected for analysis were based on three criteria:

- The record length should be of at least 50 years.
- The total missing values percentage of the record is less than 20%.
- The percentage of values assigned with quality flags per record is less than 0.1%.

Subsequently, the extracted annual maxima would be considered invalid if the missing value percentage of the year is greater than 33% and the probability of exceedance of the annual maxima concerning the rest of the record is greater than 60%. Herein, we expanded the method on three main characteristics. First, the minimum record length initially set at 50 years could now be as short as 20 years. Second, the initial upper limits of 20% missing values and 0.1% quality flags are no more (i.e., there is no limit). Third, assessing how well the temporal trend of the annual maxima time series is preserved. The rationale for the first was due to the recent surge in active stations in 2001 and to expand spatial coverage as much as possible. The rationale for the second is to avoid the exclusion of long records with large missing value percentages. That is, a station with 100 years of data, even with a 50% total missing data, could lead to an annual maxima time series of at least 50 years, which would be lost if we considered the preliminary method. These modifications to the screening method already translate into substantial differences, i.e., with the criteria by Papalexiou and Koutsoyiannis (2013)^25^, we would have ended with 13 761 station records, whereas with the new criteria proposed herein, we end with 43 585 station records. Lastly, the preliminary method’s capability to preserve the trend was not evaluated, and trend analysis would be one of the typical analyses for which HYADES will be used, so it is essential to ensure the trend is not altered.

**Fig. 4:**
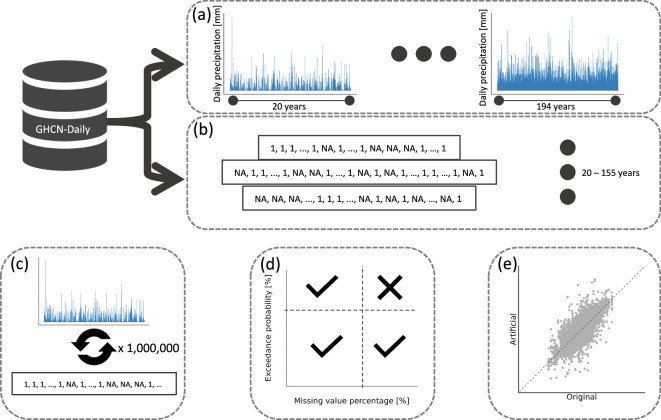
Monte Carlo simulation scheme. (**a**) extraction of all available complete (0% missing values and 0% quality flags) records from the GHCN-Daily database and generate a time series of annual maxima. (**b**) extraction of binary missing value profiles from the rest of the records. (**c**) generation of randomly altered records using the binary profiles to introduce missing values in the complete records. (**d**) generation of time series of annual maxima from the artificially created records for multiple threshold criteria values. (**e**) evaluation of the fidelity of the artificially generated annual maxima time series generated in (**d**) with the real maxima series created in (**a**).

In order to determine the new thresholds that satisfy the aforementioned criteria, we designed a scheme of Monte Carlo simulations that consisted of five steps (Fig. 4): (a) extract a subset of all available complete records in the database, (b) extract binary profiles of missing data from the rest of the records, (c) randomly alter the complete records using the binary profiles to introduce missing values, (d) generate the time series of annual maxima of the artificially created records for multiple threshold criteria values, and (e) compare the fidelity of the artificially generated annual maxima time series generated in (d) with the actual maxima series constructed from (a).

To begin, we selected only ground stations that contained consecutive records of at least 20 complete years and deleted all incomplete years or missing values around them, if any. We ended with 8 968 records with full consecutive years of lengths between 20 and 194 years. Subsequently, we generated binary missing value profiles (i.e., non-missing and missing values) of lengths between 20 and 155 years using the remaining 34 617 ground station records. To construct an artificial record with missing values, we randomly paired a complete ground station record with a binary missing value profile that, if needed, was trimmed to match the length of the initially complete record. It should be stressed that while the Monte Carlo simulation methodology is statistically grounded, the experimental design does not assume pure randomness in removing data from a time series, unlike the original approach by Papalexiou and Koutsoyiannis^25^, where a random number of missing values were distributed within a random number of years per record based on a fitted beta distribution to the percentage of years with missing values. On the other hand, in our Monte Carlo simulation design, missing values are inserted by emulating actual records with missing values. In other words, we artificially introduce real-life scenarios in which missing values within a record are present due to, for example, an instrument’s failure, damage, replacement, or maintenance.

We generated over a million different artificial records and extracted the annual maxima time series using 72 different threshold combinations with the probabilities of exceedance between 60 and 75% and the percentage of missing values between 33 and 50% using increments of 2% for both. Finally, we computed the average relative difference in mean, L-Variation, L-Skewness, L-Kurtosis, and the temporal trend between the real time series and the artificially generated ones to determine which thresholds were able to preserve most statistical properties of interest. L-moments are statistical quantities derived from the probability weighted moments (PWM) theory^26–28^. The robustness of L-moments is that they only require the random variable to have finite mean, so the L-moments exist even if the higher conventional moments do not^29,30^. The L-Variation is the L-moment analogous to the coefficient of variation and is between zero and one for a non-negative random variable. The L-Variation is a standardized measure of the dispersion of a probability distribution. L-Skewness (*τ*_3_) and L-Kurtosis (*τ*_4_) are scaled L-moments defined by

1 $${\tau }_{r}=\frac{{\lambda }_{r}}{{\lambda }_{2}},r=3,4,\ldots $$

where *λ*_*r*_ is the r-th L-moment. L-Skewness measures the asymmetry or skewness of a probability distribution. We found that the measures of shape and the trend allowed for less strict thresholds for the probability of exceedance, and the measure of dispersion and the trend allowed for less strict thresholds for the percentage of missing values. In order to preserve all the variables of interest the best, the thresholds to be used to invalidate an annual maxima value should be greater than 33% missing values within a year and greater than 60% probability of exceedance within the annual maxima time series. While we ended with the same thresholds as the preliminary method, the Monte Carlo simulations allowed us to confirm said thresholds for records of shorter lengths and no upper limit on the total percentage of missing values.

### Annual maxima time series extraction

**Fig. 5:**
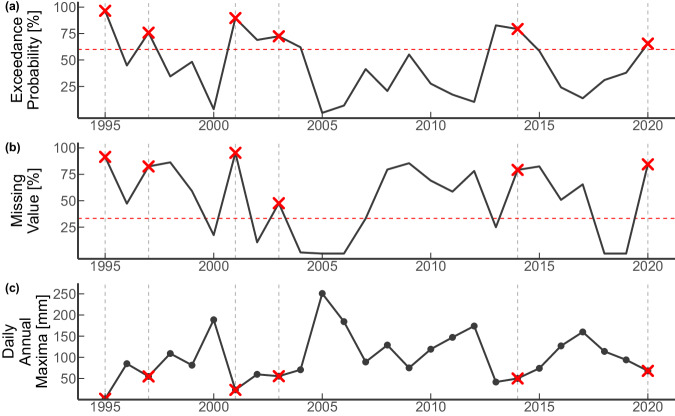
Explanatory plot of the maxima extraction method. The annual maxima of daily precipitation is extracted regardless of the quantity of missing values or quality flags as long as the record length is at least 20 years. Subsequently, the annual maxima is considered invalid (red crosses in (**c**)) if its probability of exceedance (PE%) is above 60% (red dashed line in (**a**)) and the missing-value percentage (MV%) of the year it belongs to is larger than 33% (red dashed line in (**b**)).

With the threshold criteria now defined and validated, the annual maxima time series are extracted from the 43 585 preliminary screened ground station records in four steps (Fig. 5): (a) Any quality flags (“D”, “G”, “I”, “K”, “L”, “M”, “N”, “O”, “R”, “S”, “T”, “W”, “X”, or “Z”; for details refer to https://www.ncei.noaa.gov/products/land-based-station/global-historical-climatology-network-daily) of the record are removed and considered as missing values, (b) the maximum of each year is extracted irrespective of missing values within the year, (c) the annual maxima of daily precipitation values are tested according to the threshold criteria, and if they do not fulfill them, they are considered a missing value and removed. That is, if only if a year had more than 33% of missing values and the probability of exceedance of said year’s value is greater than 60% within the maxima time series, the value extracted is considered invalid, and (d) runs of consecutive missing years as a result of (c) are removed from the annual time series. After the annual maxima of daily precipitation time series were extracted following the above steps, the total number of ground station records was reduced to 39 206, with record lengths varying between 16 and 200 years.

Note that the previous methodology described by Papalexiou and Koutsoyiannis (2013)^25^ identified and, if deemed suspicious, deleted primarily the “G” (failed gap check) and “X” (failed bounds check) quality flags, as these values could be orders of magnitude larger compared to the record’s second largest value. Herein, we automatically address all 14 quality flags by removing them because the number of introduced missing values did not impact the annual maxima time series extraction. On the contrary, we avoid spurious values previously overlooked. For example, pointing out a specific case for 1998 in Riyadh (Station id: SA000040438), the original method would report an annual maximum of 778 [mm] vs. our method, determining an annual maximum of 19 [mm]. Note that in Riyadh, between 1970 and 2010, the annual maxima of daily precipitation does not even reach 60 [mm]^31^. The discrepancy is rooted in some record entries for 1998 with the “K” flag (failed streak/frequent-value check), a flag previously overlooked by Papalexiou and Koutsoyiannis^25^.

## Data Records

The HYADES^32^ dataset consists of a station-based time series of annual maxima of daily precipitation publicly available on a Zenodo repository (https://zenodo.org/doi/10.5281/zenodo.10058983). HYADES is distributed as a single comma-separated value (CSV) file with five columns: Ground Station ID, Longitude, Latitude, Year, and Annual Maxima of Daily Precipitation in [mm]. HYADES contains a total of 39 206 ground station records with lengths varying from 16 to 200 years within the 1805–2023 period. The summary statistics of HYADES are shown in Table 1. Noteworthy, all statistical characteristics vary greatly–for instance, the mean ranges from 4.66 [mm] to 791.26 [mm], the standard deviation from 2.9 [mm] to 468.46 [mm], and the trend from −10.79 [mm/year] to 10.2 [mm/year].

**Table 1:** Summary statistics of the 39 206 stations that comprise HYADES.

	Record Length [years]	Trend [mm/year]	Median [mm]	Mean [mm]	SD [mm]	L-Scale	L-Skewness	L-Kurtosis
						*λ*_3_	*τ*_3_	*τ*_4_
Min	16	−10.79	3	4.66	2.9	1.47	−0.59	−0.2
*Q*_5_	19	−0.97	25.65	28.66	9.49	5.18	0.04	0.05
*Q*_25_	26	−0.17	36.7	40.23	15.11	8.12	0.15	0.12
*Q*_50_	40	0.03	53.8	58.3	21.18	11.28	0.22	0.17
*Q*_75_	62	0.25	76.2	81.81	31.28	16.54	0.28	0.22
*Q*_95_	100	1.09	114.3	125.14	56.97	29.92	0.38	0.31
Max	200	10.2	733	791.26	468.46	267.61	0.8	0.8
Mean	46.71	0.05	60	65.12	25.55	13.56	0.22	0.18
SD	25.9	0.74	30.36	32.65	15.46	8.12	0.1	0.08

## Technical Validation

**Fig. 6:**
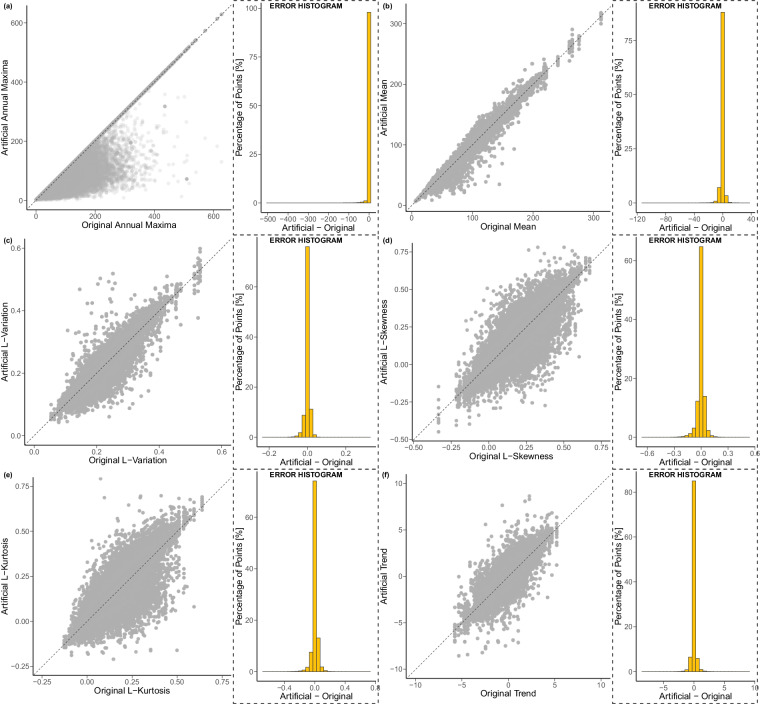
Scatter plot summary results of the 100 000 Monte Carlo simulations with artificially generated time series in the y-axis and the real time series in the x-axis. In addition, error histograms with the percentage of points in the y-axis and the error (artificially generated time series minus the real time series) in the x-axis. (**a**) Annual maxima of daily precipitation. (**b**) Mean of the annual maxima of daily precipitation. (**c**) L-Variation of the annual maxima of daily precipitation. (**d**) L-Skewness of the annual maxima of daily precipitation. (**e**) L-Kurtosis of the annual maxima of daily precipitation. (**f**) Trend of the annual maxima of daily precipitation.

To validate the HYADES extraction method to generate annual maxima of daily precipitation time series, we run once again the Monte Carlo simulation as described in the Methods section with the sole difference that now the threshold criteria was fixed to 33% percent of missing values per year and 60% probability exceedance per record. This time around, we generated 100 000 artificial records with missing values. We compared them with the entirely complete original ground station records based on the fidelity of six characteristics. The annual maxima estimates themselves as well as their statistical properties: mean, L-Variation, L-Skewness, L-Kurtosis, and temporal monotonic trend (Fig. 6). We can characterize a probability distribution’s shape, scale, location, and tail behavior through L-moments. Of particular relevance herein, the Generalized Extreme Value (GEV) distribution with probability distribution function given by

2 $${f}_{{\rm{GEV}}}(x)=\exp \left(-{\left(1+\gamma \frac{x-\alpha }{\beta }\right)}^{-1/\gamma }\right),1+\gamma \frac{x-\alpha }{\beta }\ge 0$$

allows us to estimate the tail behavior of precipitation distributions, which is crucial for understanding and predicting extreme precipitation events. Therefore, we must ensure accurate L-moment estimates, for they constitute the starting point of the aforementioned assessments.

Interpreting the scatter plots presented is straightforward and intuitive as we have the identity diagonal, and points under the diagonal show underestimation while points above it describe overestimation. Nonetheless, a basic scatter plot could be misleading because of the considerable amount of overlapping points. Thus, we added the corresponding error histograms, and now it is clear that the highest concentration of points is aligned over the identity diagonal (i.e., error equal to zero). Expressly, the properties of the HYADES time series extraction algorithm for the artificially generated records match those of the original records flawlessly most of the time. As previously mentioned, had we chosen to visualize only a scatterplot, the artificial records would falsely indicate an overall underestimation of annual maxima values (Fig. 6a). However, this alleged underestimation is nothing more than a visual artifact due to the massive overlap of points over the identity diagonal. In reality, the cloud of points below the identity diagonal represents a measly 3.27% of the points. Put another way, our method has a 96.73% success rate. The scatterplot artifact persists on the rest of the variables, but they are not as misleading because even with the visual artifact present, the results are centered around the identity diagonal. The density of points above the identity diagonal is slightly higher, indicating an overestimation of the average annual maxima (Fig. 6b). This is expected because the framework proposed deems a value invalid if it meets two conditions: the percentage of missing values within an incomplete year is higher than 33%, and the probability of exceedance is above 60%. The latter’s implication is the rejection of only small values (small in comparison with the rest of the record).

Consistent with the above findings, the most dense cloud of L-Variation points is across the identity diagonal and narrowly above it (Fig. 6c). In line with expectations for a distribution of annual maxima precipitation, the density plot for L-Skewness lies over the identity diagonal with a higher concentration of values greater than zero (Fig. 6d). L-Kurtosis measures the heaviness of the distribution’s tail relative to a normal distribution. A positive L-Kurtosis indicates a distribution with a longer right tail (i.e., extreme values on the right side of the distribution). Similar to L-Skewness, the L-Kurtosis density plot lies over the identity diagonal with a higher concentration of values greater than zero (Fig. 6e). Lastly, in the context of climate change, trend analysis helps discern transitions in precipitation patterns. We can quantify the impact of anthropogenic activities, land-use changes, and infrastructure development on precipitation regimes by assessing trends. Intriguingly, while the method does preserve the trend, it is alluring to observe that trend values are also symmetrically distributed around zero without an evident proclivity toward positive nor negative trends (Fig. 6f).

**Fig. 7:**
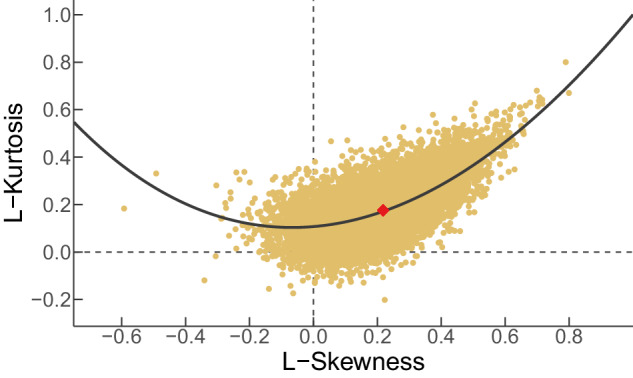
L-moments ratio diagram for the 39 206 HYADES records and the theoretical line of the Generalized Extreme Value distribution. L-Kurtosis versus L-Skewness points in matte yellow. Average point in red.

To this point, we have determined a method to extract the annual maxima of daily precipitation and have demonstrated that its success rate is circa 97%. Consequently, it is safe to move forward in the analysis and illustrate one of the primary analyses for which the HYADES data set will be useful: distribution fitting. The method of L-moment ratio diagrams is a powerful approach for fitting probability distributions^33^. Calculating the ratios of L-moments stabilizes the estimation process and enhances the reliability of distribution fitting^34^. These ratios are often more robust and less sensitive to sample size variations than individual L-moments, making them particularly useful for regions with limited data^35^. In essence, the L-moment ratio diagrams present a graphical comparison between observed L-ratio values and points, lines, or areas formed by parametric distributions’ theoretical formulas^36^. The L-Kurtosis versus L-Skewness diagram reveals that the cloud of points corresponding to the L-moments of the 39 206 ground station records that constitute the HYADES data set resembles the theoretical GEV curve (Fig. 7). Furthermore, the point that marks the average L-moment values falls precisely on the curve with a shape parameter of *γ* ≈ 0.1 indicating a prevalence of the Fréchet law^25^.

## Data Availability

Pre- and post-processing code was written in R, and it is publicly available at https://github.com/MiRoVaGo/hyades.
